# Rationale and design of the Caloric Restriction and Exercise protection from Anthracycline Toxic Effects (CREATE) study: a 3-arm parallel group phase II randomized controlled trial in early breast cancer

**DOI:** 10.1186/s12885-018-4778-7

**Published:** 2018-09-03

**Authors:** Amy A. Kirkham, D. Ian Paterson, Carla M. Prado, John M. Mackey, Kerry S. Courneya, Edith Pituskin, Richard B. Thompson

**Affiliations:** 1 Department of Biomedical Engineering, University of Alberta, 1098 Research Transition Facility, 8308-114 Street, Edmonton, AB T6G 2V2 Canada; 2 Department of Medicine, Division of Cardiology, University of Alberta, Edmonton, Canada; 3 Department of Agricultural, Food & Nutrition Science, University of Alberta, Edmonton, Canada; 4 Department of Oncology, University of Alberta, Edmonton, Canada; 5 Faculty of Kinesiology, Sport, and Recreation, University of Alberta, Edmonton, Canada; 6 Faculty of Nursing, University of Alberta, Edmonton, Canada

**Keywords:** Anthracyclines, Cardiotoxicity, Breast cancer, Exercise, Caloric restriction, Magnetic resonance imaging

## Abstract

**Background:**

Anthracycline chemotherapy agents are commonly used to treat breast cancer, but also result in cardiac injury, and potentially detrimental effects to vascular and skeletal muscle. Preclinical evidence demonstrates that exercise and caloric restriction can independently reduce anthracycline-related injury to the heart as well as cancer progression, and may be promising short-term strategies prior to treatment administration. For women with breast cancer, a short-term strategy may be more feasible and appealing, as maintaining regular exercise training or a diet throughout chemotherapy can be challenging due to treatment symptoms and psychosocial distress.

**Methods:**

The Caloric Restriction and Exercise protection from Anthracycline Toxic Effects (CREATE) study will determine whether acute application of these interventions shortly prior to receipt of each treatment can reduce anthracycline-related toxicity to the heart, aorta, and skeletal muscle. Fifty-six women with early stage breast cancer scheduled to receive anthracycline treatment will be randomly assigned to one of three groups who will: 1) perform a single, 30-min, vigorous-intensity, aerobic exercise session 24 h prior to each anthracycline treatment; 2) consume a prepared diet reduced to 50% of caloric needs for 48 h prior to each anthracycline treatment; or 3) receive usual cancer care. The primary outcome is magnetic resonance imaging (MRI) derived left ventricular ejection fraction reserve (peak exercise LVEF – resting LVEF) at the end of anthracycline treatment. Secondary outcomes include MRI-derived measures of cardiac, aortic and skeletal muscle structure and function, circulating NT-proBNP, cardiorespiratory fitness and treatment symptoms. Exploratory outcomes include quality of life, fatigue, tumor size (only in neoadjuvant patients), oxidative stress and antioxidants, as well as clinical cardiac or cancer outcomes. MRI, exercise tests, and questionnaires will be administered before, 2–3 weeks after the last anthracycline treatment, and one-year follow-up.

**Discussion:**

The proposed lifestyle interventions are accessible, low cost, drug-free potential methods for mitigating anthracycline-related toxicity. Reduced toxic effects on the heart, aorta and muscle are very likely to translate to short and long-term cardiovascular health benefits, including enhanced resilience to the effects of subsequent cancer treatment (e.g., radiation, trastuzumab) aging, and infection.

**Trial registration:**

ClinicalTrials.gov NCT03131024; 4/21/18.

## Background

Anthracycline chemotherapy agents are one of the most widely used and highly active classes of anticancer therapies for numerous cancer types, including breast, lymphoma, leukemia, uterine, ovarian, and lung cancers [1, 2]. However, anthracyclines are associated with cardiomyocyte damage that may be immediate, cumulative, and synergistic with other cancer treatments [3, 4]. This damage manifests as cardiotoxicity, which is diagnosed by a > 10 percentage point drop in left ventricular ejection fraction (LVEF) from baseline to a LVEF < 53% [5]. A recent prospective study reported a 10% incidence of anthracycline-related cardiotoxicity among patients with breast cancer within the first year after treatment [6]. However, prevalence of subclinical cardiotoxicity, which could be defined by elevated circulating cardiac biomarkers [7] or deterioration in left ventricular (LV) longitudinal strain [8], is likely substantially higher [9]. Subclinical cardiotoxicity may increase susceptibility to future insults, including subsequent receipt of other cardiotoxic cancer therapies, aging, and infection [9].

Anthracycline treatment, in addition to other cardiotoxic breast cancer therapies (i.e. left breast radiation, trastuzumab) play a direct role in the increased risk of cardiovascular disease and related death among women who have had a breast cancer diagnosis relative to those who have not [10–12]. Strategies to reduce anthracycline-related cardiac injury are required to balance the oncologic efficacy with cardiac safety of breast cancer therapy. To date, these have included dose minimization/reduction [9], which may translate to reduced oncological benefit [13], or traditional cardiovascular pharmacological agents [14–16], which can be associated with additional side effects and inadequate adherence.

Lifestyle interventions including exercise training and diet modification are novel non-pharmacological potential cardio-protective therapies. Firstly, exercise training is widely established to ameliorate anthracycline-related cardiac injury in preclinical studies, and cardiovascular risk factors in breast cancer survivors [17]. However, during chemotherapy treatment, breast cancer patients have modest and variable adherence to exercise 3×/week due to treatment symptoms, cancer-related appointments, and travel to the exercise facility [18]. Secondly, nutrient deprivation increases the resistance of healthy cells, but not cancer cells, to the damaging effects of oxidative stress by 1000-fold [19, 20]. This phenomenon, termed ‘selective stress resistance’ could dramatically reduce treatment toxicity. For example, 40 days of a 35% calorie restricted diet led to 100% protection from anthracycline-related cardiotoxicity and death in a preclinical model [21]. However, this type of long-term caloric restriction regimen is likely unfeasible for individuals during chemotherapy as they typically suffer from malnutrition, psychological distress, and other treatment complications [22]. Therefore, given that potential for universally high adherence among all patients is important for interventions to achieve far-reaching effects, more feasible methods of lifestyle intervention are required during chemotherapy for breast cancer.

Short-term administration of exercise and caloric restriction shortly prior to receipt of anthracycline treatment capitalizes on the potentially critical, yet underappreciated protective factor of intervention timing, and may ameliorate the issue of inadequate adherence to longer interventions. Based on preclinical evidence [23, 24], we previously investigated the effect of 30 min of vigorous-intensity treadmill walking performed 24 h prior to every anthracycline treatment (i.e., one session per 2–3 week cycle) on markers of cardiotoxicity and treatment symptoms in women with breast cancer [25, 26]. This is a highly feasible approach as patients are usually scheduled to attend the treatment center for blood tests and physician examination the day prior to treatments and this day would also correspond with the nadir of symptoms from the previous treatment. We found that, in contrast to exercise training during chemotherapy, there was 100% adherence to the acute intervention, and no adverse events. Further, the exercise session significantly attenuated NT-proBNP at 24–48 h after the first treatment, which is a circulating biomarker of anthracycline-related cardiac damage and cardiac events. At treatment completion, performing the session prior to each therapy had prevented an increase in resting heart rate (highly prognostic of mortality) and cardiac output, and reduced body weight and prevalence of sore muscles, low back pain and depressed mood relative to usual care [25, 26].

Preclinical studies have reported that 1–2 months of mild caloric restriction or short periods (24–60 h) of more severe caloric restriction or fasting may be effective to prevent cardiac and other toxicities from supraclinical chemotherapy doses [20, 22, 27]. While the effect of caloric restriction on chemotherapy-related side effects has not been studied in women with breast cancer, a pilot study of a complete fast for 24 h prior to and 24 h after anthracycline treatment was well tolerated and reduced hematological toxicity, but cardiovascular function was not measured [28]. Based on this available evidence, 48 h of 50% caloric restriction is likely to provide protection against the much lower clinical doses given each treatment cycle for humans and be well tolerated and safe for women receiving chemotherapy for breast cancer. Overall, there is promising preliminary evidence that acute exercise or caloric restriction could be feasible and effective methods to reduce anthracycline-related cardiotoxicity and treatment symptoms.

Preclinical evidence suggests anthracycline-related toxicity is not confined to the heart, but similarly affects the vessels and skeletal muscle [29]. Available studies in humans are limited in depth and methodology, and lack an integrative approach to characterizing all of these related tissues in the same patients. Considering the established role of vascular and skeletal muscle pathophysiology in traditional heart disease [30, 31], these treatment toxicities likely play a role in the increased risk of cardiovascular disease and exercise intolerance, as well as experience of fatigue, reduced quality of life, and overall health among breast cancer survivors. Both exercise and caloric restriction have the potential to modify anthracycline-related vascular and skeletal muscle dysfunction via modulation of inflammation, cellular apoptosis [32–34], oxidative stress, and endothelium-dependent vasodilation (i.e., nitric oxide bioavailability) [35, 36].

Additionally, both caloric restriction and exercise have antineoplastic effects and can potentially sensitize the tumor to chemotherapy [22, 37], further reducing mortality risk in individuals diagnosed with cancer. Although substantial epidemiological evidence has established the relationship between physical activity and cancer prevention and recurrence [38], experimental evidence exists primarily in preclinical models. Likewise, caloric restriction has been widely established to suppress carcinogenesis in over 40 preclinical studies, yet experimental evidence in human cancer patients is limited [39]. These interventions could also be hypothesized to have positive effects on fatigue, chemotherapy symptoms, and health-related quality of life, given the potential benefit on organ systems integral to health.

The Caloric Restriction and Exercise protection from Anthracycline Toxic Effects (CREATE) study aims to determine if short-term application of aerobic exercise or caloric restriction prior to each anthracycline chemotherapy treatment for breast cancer will reduce the detrimental effects of anthracyclines on the heart, vessels and skeletal muscle and treatment symptoms. The exploratory aims are to determine the effect of the interventions on tumor size, quality of life, fatigue, oxidative stress, and clinical cardiac and cancer outcomes.

## Methods

### Design and ethics

**Fig. 1:**
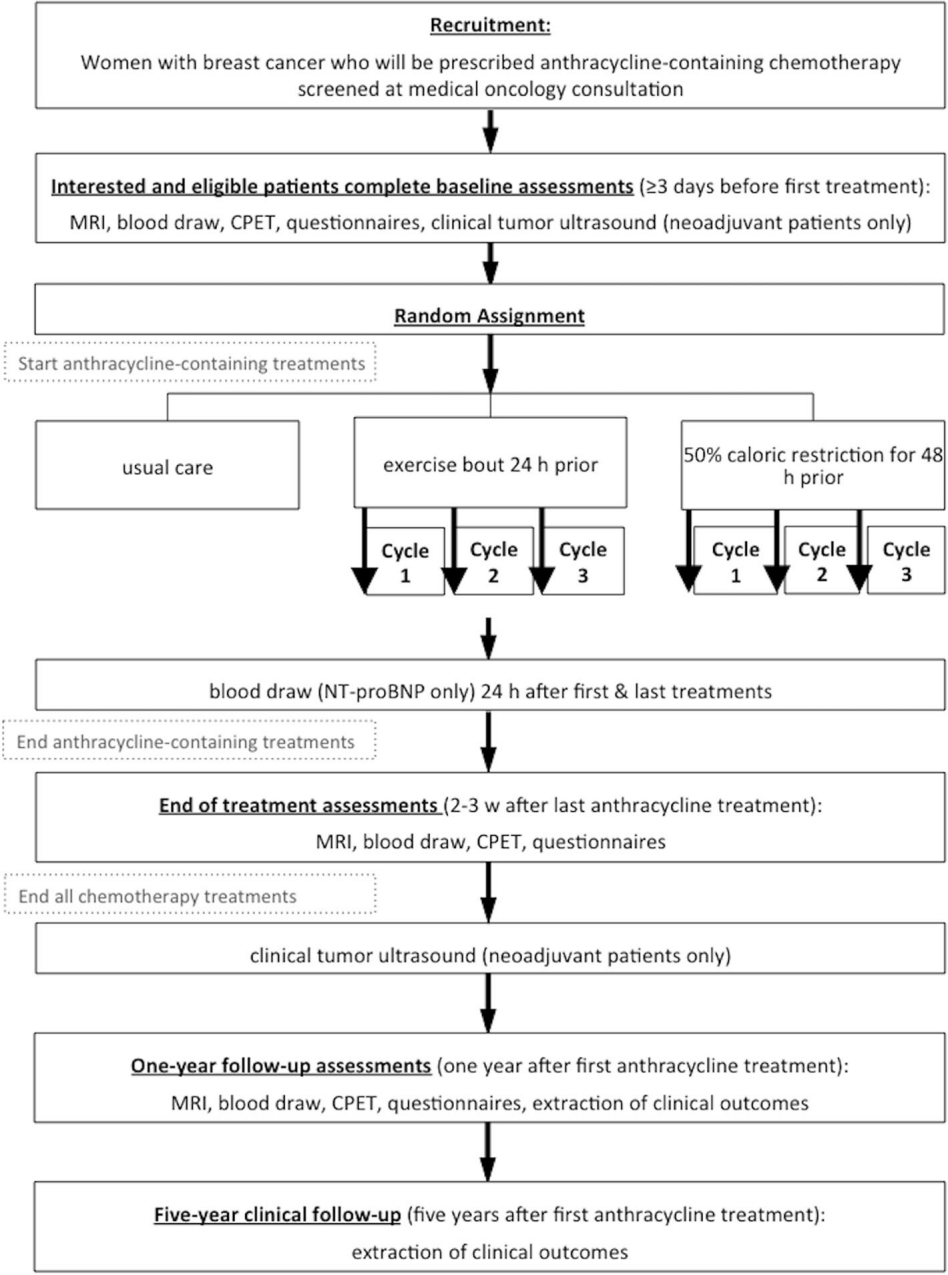
Study design. Note: Most patients at the Cross Cancer Institute will receive three anthracycline cycles total, followed by additional chemotherapy treatments (typically taxanes). In the case where patients receive more than three anthracycline treatments, the intervention will be applied to all cycles received

The CREATE study is a three-arm, parallel group phase II randomized controlled trial that will compare the effects of a single aerobic exercise session performed 24 h prior to each anthracycline treatment, to 50% caloric restriction for 48 h prior to each anthracycline treatment, to usual care on cardiac, vascular, and skeletal muscle changes in women with breast cancer. The overall study design is summarized in Fig. 1. The Health Research Ethics Board of Alberta (HREBA) Cancer Committee approved this study and all participants will provide written informed consent. Confidentiality of potential and enrolled participants will be protected by access being restricted to study staff only. All protocol amendments will be approved by the HREBA and updated on clinicaltrials.gov [40].

### Randomization

Randomization will consist of a permutated block design with random block sizes of six and nine and stratification by two factors with a 1:1:1 allocation ratio. The stratification factors will include: 1) receipt of trastuzumab (yes/no), as it is also known to affect cardiac function; and 2) adjuvant/neoadjuvant protocol to ensure an equal distribution of neoadjuvant patients to assess the exploratory tumor size outcome. An external party to the study will generate the randomization sequence using a spreadsheet random function. This same individual will put the group assignment into sequentially numbered, sealed, opaque envelopes. Randomization will occur following completion of baseline assessments.

### Participants

Potential participants will be identified and screened at new breast cancer patient medical oncology clinics at the Cross Cancer Institute in Edmonton, Alberta, Canada, the largest cancer treatment center serving Northern Alberta. Eligibility criteria are listed in Table 1. Participants will sign the consent form with and be assigned to a group by the same investigator. Participants will be reimbursed for parking for all required study visits but there is no other compensation for participation.

**Table 1:** Participant eligibility criteria

Inclusion criteria	Exclusion criteria
• ≥ 18 years of age • Female • Able to read and communicate in English • Diagnosis of stage IA-IIIC breast cancer • Scheduled to receive anthracycline-containing chemotherapy • Willing and able to adhere to either intervention	• Contraindications to research MRI (e.g. pacemaker, magnetic implant) • Contraindications to maximal exercise testing • Pregnancy • Limitations to sustained exercise on all potential modes (i.e., treadmill, elliptical, bike) • Body mass index <19 kg/m^2^ or history of eating disorder • Diabetes • Severe food allergies or diet restrictions

### Outcome measures

The study outcome measures and their timing are summarized in Table 2. The primary outcome measure is cardiac function reserve capacity (LVEF reserve), which is calculated as peak exercise LVEF – resting LVEF, at the end of anthracycline treatment relative to the control group. Despite being the primary parameter used to diagnose cardiotoxicity, it is recognized that resting LVEF is insensitive to subclinical anthracycline-related cardiac damage due to the heart’s capacity to compensate for dysfunction at rest. By the time a decrease in resting LVEF is realized, there is likely already substantial and irreversible damage to the myocardium [3]. In women previously treated for breast cancer, a systematic review demonstrated that the application of either exercise or pharmacological stress during cardiac imaging techniques identified cases of cardiac dysfunction that were not identified using resting imaging [41]. More specifically, in women receiving anthracycline treatment for breast cancer, LVEF reserve (peak – rest) and peak LVEF after two treatments were significant predictors of a resting LVEF drop to < 50% at 18 months after treatment [42].

**Table 2:** Summary of study outcome measures

Measure	Assessment Method	72+ h pre-anth	24 h post 1st & last anth	2–3 w post last anth	1 y post 1st anth	5 y post 1st anth
Primary Outcome:						
LVEF reserve (peak – rest)	MRI	x		x	x	
Secondary Cardiac Outcomes:						
Peak and resting LVEF	MRI	x		x	x	
Resting and peak LV strain	MRI	x		x	x	
LV volumes and cardiac output	MRI	x		x	x	
Myocardial T1	MRI	x		x	x	
LV mass	MRI	x		x	x	
NT-proBNP	Venipuncture	x	x	x		
Secondary Vascular Outcome:						
Ascending and descending aortic distensibility	MRI	x		x	x	
Secondary Skeletal Muscle Outcomes:						
Lower leg oxygen extraction	MRI	x		x	x	
Lower leg oxygen consumption	MRI	x		x	x	
Thigh muscle mass	MRI	x		x	x	
Thigh muscle quality	MRI	x		x	x	
Secondary Integrative Outcomes:						
Whole body oxygen consumption reserve	CPET	x		x	x	
Treatment symptoms	Rotterdam Symptom Checklist	x		x	x	
Exploratory Outcomes:						
Tumor size	Ultrasound	x		x^a^		
Quality of life	FACT-General questionnaire	x		x	x	
Fatigue	FACT-Fatigue questionnaire	x		x	x	
Markers of oxidative stress/antioxidants	Venipuncture	x		x		
Clinical Outcomes					x	x
Safety Outcomes:						
Clinically reported symptoms	ESAS	Will be extracted from clinical records for intervention period				
Intervention-related symptoms or adverse events	Food diary, Exercise session log	Will be collected for each intervention delivered				

*anth* anthracycline chemotherapy treatment, *CPET* cardiopulmonary exercise test, *ESAS* Edmonton Symptom Assessment System, *LVEF* left ventricular ejection fraction

^a^Typically measured before and after completion of all chemotherapy as standard of care

Secondary outcome measures include other parameters of cardiac function and structure (LV resting and peak strain, myocardial T1, LV volumes and mass, resting and peak cardiac output, peak LVEF, resting LVEF) and aortic distensibility at the end of anthracycline treatment, as well as circulating NT-proBNP at 24 h post the first and last treatment. Secondary skeletal muscle outcome measures include lower leg oxygen consumption and extraction, and thigh mass and quality at the end of anthracycline treatment. Secondary integrative outcome measures include whole body oxygen consumption reserve and treatment symptoms. Both interventions are hypothesized to reduce toxic effects on the heart, aorta, skeletal muscle and integrative measures relative to usual care.

A number of additional outcome measures of interest will be collected that are designated as exploratory. Exploratory outcomes at end of treatment include: 1) tumor size in neoadjuvant patients; 2) patient-reported quality of life and fatigue; 3) circulating markers of oxidative stress and antioxidants. To assess for potential longer-term effects of the interventions, a one-year follow-up with assessment of all MRI and questionnaire measures as well as CPETs and clinical outcomes (e.g. cancer recurrence, cardiac events, hospitalizations, mortality) will be completed. These combined clinical outcomes will also be collected as exploratory outcomes after five years of follow-up.

Safety outcomes will include clinically reported Edmonton Symptom Assessment scores throughout the intervention period as well as intervention-related symptoms or adverse events collected via food diaries and supervised exercise session monitoring.

#### Exercise MRI

Physiologic reserve refers to capacity for augmented function, beyond resting function, in organs and biological systems. A gradual decline in physiologic reserve of all tissues and organ systems is a hallmark of aging and many chronic diseases [43, 44]. Chemotherapy has the potential to rapidly accelerate this decline. For example, it has been reported that the cardiorespiratory fitness reserve capacity of one-third of breast cancer survivors is less than that required for independent living during or after treatment [45]. The proposed MRI-derived measures in the current study utilize this concept of using exercise stress to assess change in physiologic reserve capacity of the heart and skeletal muscle.

**Fig. 2:**
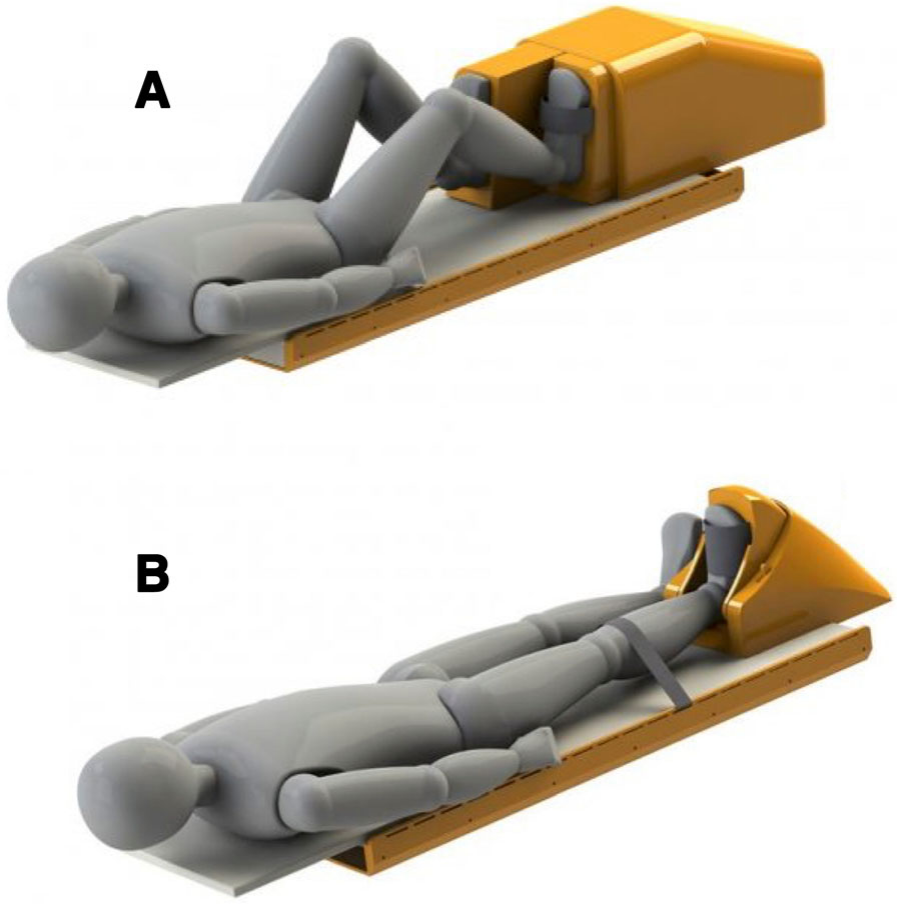
Ergospect ergometers made for use inside MRI scanner bore for whole body exercise (**a**) and isolated calf skeletal muscle exercise (**b**)

Cardiac, aortic, and skeletal muscle structure and function will be assessed via MRI (Prisma 3.0 Tesla, Siemens, Erlangen, Germany) at the Peter S. Allen MR Research Centre (Edmonton, Alberta) and custom analysis with Matlab software (Mathworks Inc., Natick, MA). Cardiac and muscle reserve function will be assessed using a commercially available, MRI-compatible ergometer with two modules designed for exercise testing within the scanner bore (Ergospect, Innsbruck, Austria) (Fig. 2). The ‘Cardiostep module’ is a horizontal resisted stepping device (Fig. 2A) that can be used for whole body exercise to increase central hemodynamics and augment cardiac function. The ‘Trispect module’ is a resisted plantar flexion (i.e., toe pointing) device (Fig. 2B) that can be used to exercise the muscle of one lower leg to allow for evaluation of skeletal muscle function independent of cardiac function.

#### MRI protocol

**Fig. 3:**
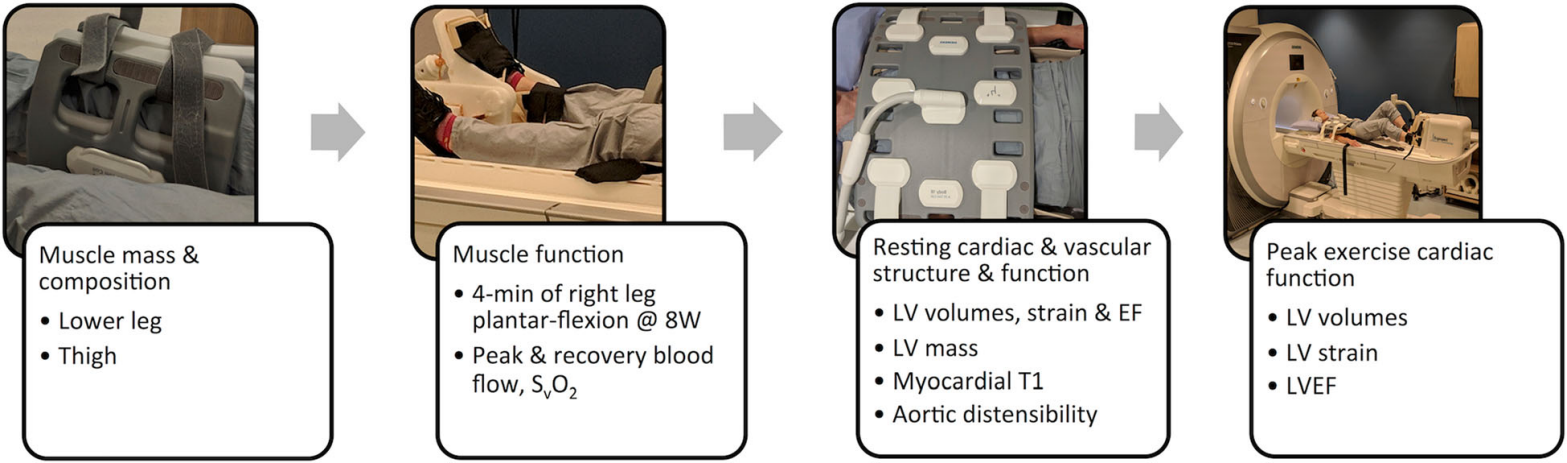
MRI protocol

The MRI protocol is summarized in Fig. 3. The MRI protocol starts with quantification of skeletal muscle mass of the right lower leg and thigh using a Dixon fat-water separation acquisition method [46] that enables precise and reproducible segmentation of skeletal muscle and adipose tissue. Next, time-resolved phase contrast and oxygen concentration sensitive images were acquired to quantify resting blood flow and venous oxygen saturation (S_v_O_2_), respectively, in the superficial femoral vein of the right leg, similar to that previously described [47]. S_v_O_2_ is quantified via susceptometry-based oximetry using deoxyhemoglobin as an intrinsic contrast agent. Together with arterial oxygen saturation and measured hemoglobin concentration, the volume of oxygen consumption (VO_2_) can be calculated for the right lower leg skeletal muscle, indexed to lower leg muscle mass (mL/min/kg), as: lower leg VO_2_ = blood flow*(S_a_O_2_ – S_v_O_2_)* [Hb]*Ca; where: S_a_O_2_ = arterial oxygen saturation from pulse oximeter, [Hb] = hemoglobin concentration, Ca = hemoglobin oxygen carrying capacity (1.34 mL O_2_/g Hb). Hemoglobin concentration corresponding to pre- and post-anthracycline treatment will be extracted from clinical records. If no recent clinical measure of hemoglobin is available for the one-year follow-up assessment then it will be measured as part of the study. Following resting evaluation, the right leg performs plantar flexion exercise on the Trispect module for four minutes at 8 watts. This wattage was chosen as it was equivalent to 60% of the average peak wattage attained in pilot testing of a maximal incremental plantar flexion test in *n* = 20 of the same population. Heart rate and rating of perceived exertion will be recorded every minute using an index finger pulse oximeter and self-report, respectively, while brachial blood pressure will be measured every two minutes. Flow and S_v_O_2_ imaging, identical to the resting evaluation, will commence immediately upon discontinuation of exercise to capture the peak and four minutes of recovery data for blood flow and S_v_O_2_. Images are repeated every 3.4 s.

Following 10 min of rest, resting cardiac and vascular structure and function are assessed. ECG-gated, end-expiration breath-hold techniques will be used to acquire single cardiac cycle transverse images of the ascending and descending aorta. Brachial pulse pressure will be assessed twice, 60 s apart at the time of aortic imaging to calculate aortic distensbility as change in cross-sectional area of the aorta divided by pulse pressure. Myocardial T1 mapping will be acquired in two mid-ventricular short axis slices using both SASHA [48] and MOLLI [49] approaches. A free-breathing real-time cine imaging approach will be used to acquire parallel short-axis slices throughout the LV, as well as 4- and 2-chamber long axis images [50]. Full coverage of the LV long and short axis is achieved in ~ 45 s. This technique is ideal for exercise as it does not require ECG gating and removes the requirement for breath holding during image acquisition. The Cardiostep exercise protocol will start at 20 watts and increase by 5 watts every 15 s until volitional exhaustion. The exercise will take place within the scanner bore with a study staff member inside the scanner room to encourage the participant and record a rating of perceived exertion (0–10) every minute. Acquisitions will be performed at rest and immediately upon attainment of peak exercise. LVEF will be calculated as: (end-diastolic volume – end-systolic volume) / end-diastolic volume, using custom post-processing Matlab software.

#### Cardiopulmonary exercise testing

The integrated effect of impaired reserve function in the heart, vascular, and skeletal muscle systems is reduced cardiorespiratory fitness, measured as peak whole body oxygen consumption reserve (VO_2_R). VO_2_R will be measured using the gold standard cardiopulmonary exercise test (CPET). A modified Balke or Bruce treadmill protocol will be utilized until volitional exhaustion or other indications for termination [51]. Choice of protocol is based upon baseline fitness and simultaneous enrolment in an observational study using the Bruce protocol; and will be kept consistent within a given participant over time. Continuous expired gases and heart rate will be measured with a calibrated TrueOne 2400 metabolic cart (Parvo Medics, Sandy, UT). The highest volume of oxygen consumed during a 20-s period minus resting oxygen consumption will be taken as VO_2_R. The test will be performed according to the American College of Sports Medicine guidelines by an exercise physiologist [51]. The CPET will be performed on a separate day from the MRI or on the same day but with at least one hour of rest after completion of the MRI scan. Similar timing between MRI and CPET will be used for follow-up assessments for each participant. The baseline CPET will be performed at least 72 h in advance of the first treatment.

#### Circulating biomarkers

Levels of the N-terminal prohormone of BNP (NT-proBNP) after the first anthracycline treatment, as well as persistent elevations at 24 h after multiple treatments are predictive of a significant reduction of LVEF over time [52, 53]. NT-proBNP will be assessed at study enrolment, at 24–28 h after the first and last anthracycline treatment, as well as end of anthracycline treatment. Venipunctures will always be performed prior to the MRI and CPET such that results will not be influenced by exercise. NT-proBNP will be assessed in serum via electrochemiluminescence sandwich immunoassays (Roche Diagnostics, Laval, Quebec) by an accredited hospital laboratory.

A reduction in oxidative stress and increase in antioxidants are the primary mechanism hypothesized in exercise cardio-protection [17]. As an exploratory outcome, serum from study enrolment and end of treatment will also be stored at − 80° at the Canadian Biospecimen Repository (Edmonton, Alberta) for batch analysis of markers of oxidative stress and antioxidants. Specific assays/markers will be chosen upon completion of the study to ensure use of the most modern assays.

#### Questionnaires

Patient-reported outcomes will be assessed with instruments validated for use in cancer populations. Quality of life and fatigue will be assessed by the Functional Assessment of Cancer Therapy (FACT)– General and FACT-Fatigue [54], respectively. Treatment symptoms will be assessed by the Rotterdam Symptom Checklist [55].

#### Clinical data

Clinical data that will be extracted from patient records for study use will include ultrasound-assessed tumor size (in neoadjuvant patients only), anthracycline dose received, relative dose intensity, Edmonton Symptom Assessment System responses, hemoglobin, hematocrit, other prescribed drugs, cancer or cardiac events, hospitalizations, and mortality. This data will be collected for participants who discontinue or deviate from intervention protocols.

#### Data management

All research data will be de-identified using a random study ID, and will be securely stored with access provided to study staff and investigators only. All data that requires electronic entry will be double-checked for accuracy.

### Interventions

Caloric restriction intervention: Participants who are randomized to the caloric restriction arm will receive 48 h worth of prepared meals consisting of 50% of their daily caloric requirement. Caloric needs will be measured using resting energy expenditure (REE) assessed at baseline using indirect calorimetry in the morning after a 10–12 h water-only fast. Total daily energy expenditure will be calculated as REE multiplied by 1.4, which accounts for a low level of physical activity [56]. Participants will choose three isocaloric meals and two snacks consisting of half of the calories provided in the meals from a prepared menu with several options. Food will be freshly prepared within a day of the intervention for each participant at a metabolic kitchen, with each ingredient weighed to one-tenth of a gram. Each meal and snack will consist of North American macronutrient composition guidelines (30% protein, 20% fat, 50% carbohydrates) [57] calculated using Food Processor SQL (v11.0.3; ESHA Research, Salem, OR), and will be individually packaged and labelled. All food to be consumed within the 48 h period will be packed into an insulated cooler to be picked up by or delivered to the participant. To track adherence and adverse events, participants will be asked to keep a food diary to record any dietary deviations, as well as whether they experience any adverse nutrition impact symptoms (e.g., light-headedness, dizziness, nausea, diarrhoea).

Exercise intervention: Participants who are randomized to the exercise arm will perform a single bout of supervised aerobic exercise scheduled such that it would end approximately 24 h prior (window of 22–26 h allowed) to each of their scheduled anthracycline treatment times. The session will consist of a 10-min warm-up, 30 min performed at 70–75% of measured heart rate reserve, which corresponds to a vigorous intensity [51], followed by a 5-min cool-down. As an adjunct method of exercise prescription, the intensity will be adjusted as needed to maintain a minimum rating of perceived exertion (Borg 6–20 scale) of 13 (“somewhat hard”) and a maximum of 17 (“very hard”). An exercise physiologist will supervise the session one-on-one and will document any exercise-related symptoms or major adverse events.

Usual care: As there are currently no clinical guidelines or recommendations on exercise timing or calorie reduction in relation to chemotherapy treatment, standard oncology care recommendations to maintain a pre-diagnosis exercise routine and diet throughout treatment was chosen as the comparator. Participants in this group will receive a phone call from study staff during each anthracycline treatment cycle to maintain engagement and enhance retention.

There may be potential for the two interventions to have a synergistic effect on our outcomes if they were combined. However, evaluating the independent impact, safety and efficacy of a caloric restriction versus an exercise intervention in humans is essential prior to combining these interventions. All participants will be asked to restrict any exercise performed on their own to light to moderate intensity for the 72 h prior and also for 48 h after each anthracycline treatment to isolate the effect of the pre-treatment exercise intervention. Compliance with this restriction will be assessed using a modified version of the Godin Leisure Time Exercise Questionnaire [58] for each treatment. Overall physical activity and dietary intake for the duration of the intervention period (~ 3 months) will be quantified and compared between groups by self-report using the Recent Physical Activity Questionnaire [59] and the Block Brief 2000 Food Frequency Questionnaire (Nutrition Quest, Berkley, CA). These questionnaires will also be administered at study entry and one-year follow-up with reference to their habits over the previous three months.

### Blinding

It will not be possible to blind participants or interventionists to their group assignment. It is unlikely that the lack of blinding will influence the primary and secondary physiological outcomes but it could potentially influence the patient-reported outcomes. Study staff that are blinded to group assignment and prior test results will perform CPETs and circulating biomarker analyses. All MRI analyses (i.e. the quantification of data from the images) where user input can impact data (e.g., LV volume tracing) will be blinded to group assignment and time point using random numbers.

### Sample size determination

There is no available data on the effect of short-term caloric restriction or exercise on LVEF reserve. In the pilot study of the exercise intervention there was a large effect size (Cohen’s f = 0.454) on NT-proBNP. Based on this and the sensitivity of using both MRI and exercise to elucidate subclinical dysfunction, we expect at least a medium effect size for the primary outcome in the current study (Cohen’s f = 0.25). Using *n* = 15 sample size per group for a three-arm, three-repeated measures design, there is > 90% power to detect a medium effect size at *p* = 0.05 (G*Power Version 3.0.10). This sample size is further substantiated by the finding that a 3 percentage point change in resting LVEF can be detected with cardiac MRI with n = 15 patients, an 85% reduction in the sample size required to detect the same change using echocardiography [60]. The primary outcome in the current study, LVEF reserve is expected to be more sensitive than resting LVEF and therefore this sample size is expected to detect a difference between the intervention groups and the control group. A total of *n* = 56 patients (*n* = 18–19 per group) will be enrolled to allow for a 20% rate for dropout, death, and technical difficulties.

### Statistical analyses

Given the longitudinal study design, a linear mixed model analysis that includes both fixed and random effects will be used. The repeated measures on a single subject result in correlated outcome data, and the random effects allow this correlation to be explicitly modeled. An additional advantage of this model is that it allows for missing data on a subject without deleting all the data for that subject; as such imputation for missing data will not be performed. The model also allows for covariates to be tested and can include time varying covariates (e.g. treatments received post anthracyclines). One assumption of mixed models is that the residuals from the model are normally distributed. If that is not the case, a Generalized Linear Mixed model that can fit other distributions will be used. All analyses will be performed using both intention-to-treat and per protocol approaches. Although it is hypothesized is that each intervention will be effective relative to usual care, there is not a strong rationale to expect a difference in efficacy between the two interventions. However, each group will be compared against the remaining two groups to determine whether one lifestyle intervention is more efficacious or whether patient preference and tolerance can determine choice of intervention.

### Data monitoring and adverse events

A data monitoring committee is not needed due to the low risks associated with the interventions. The diet intervention group participants report symptoms or adverse events on their food diary, while the supervising exercise physiologist will document occurrence of the same during the exercise intervention. Intervention participants are also asked to contact study staff if any adverse events potentially related to the interventions occur beyond the intervention period. Serious adverse events will be reported to the study doctors and the ethics board. A statistician not associated with the study will perform an interim analysis of resting LVEF, as this is the standard parameter used to monitor cardiotoxicity within oncology practice. Resting LVEF will be compared between blinded groups after completion of the end of treatment assessment for the first thirty participants. These results will be unblinded, and along with documentation of adverse events, be provided to the study doctors who may make the decision to stop the study early if there is an increased risk or benefit deemed to be of clinical relevance at this time point. No auditing of trial conduct is planned. Participants who suffer harm from trial participation will be offered care by the study physicians.

### Dissemination

A lay summary of study results will be provided to all study participants. The study results will be published in a peer-reviewed clinical journal. The study sponsor will disseminate the study results to their stakeholders.

## Discussion

Among women diagnosed with breast cancer, anthracycline chemotherapy-related cardiac injury is a dose-limiting side effect, a distressing long-term health concern, and a likely contributor to increased cardiovascular disease risk. The current study proposes two acute lifestyle interventions that have the potential to reduce cardiovascular toxicity, thereby challenging the current paradigm of drug-related management of breast cancer therapy side effects. As a crucial requirement for widespread impact, these interventions have the potential for high adherence and uptake by the wide majority of patients of all demographics, geographic locations, and health statuses, and are low cost and scalable as part of supportive cancer care. Reduction in the amount of anthracycline-related damage to the heart, aorta and muscle is very likely to translate to both short and long-term improvements in cardiovascular health, and enhance resilience to the effects of aging, infection, and future cancer treatment on the cardiovascular system. Importantly, the interventions empower women receiving chemotherapy for breast cancer to play a role in their own treatment process and future health. If our hypotheses regarding exploratory objectives are correct, these interventions may also have additional benefits, including improving tumor response to chemotherapy and improving health-related quality of life, and fatigue. Furthermore, through the use of novel and comprehensive MRI techniques, this study will contribute new knowledge regarding the effects of anthracyclines on cardiac reserve, vascular function, and skeletal muscle and their integrative effects, including their relationship with cardiorespiratory fitness and patient-reported outcomes.

This study design is not without limitations or practical issues. Baseline assessments must be completed at least 72 h prior to the first anthracycline treatment to allow enough time for randomization, and upon assignment to the caloric restriction group, REE assessment and preparation and delivery of the diet by 48 h prior to the first treatment. Recruitment of participants will take place at the medical oncology consultation and patients will typically start anthracycline treatment the following week. Some patients require further testing to determine treatment course (and therefore eligibility) and will then start treatment very quickly upon receipt of test results. Therefore some eligible patients may not be able to be enrolled due to timing constraints.

While the sample size is calculated based on the sensitivity of our primary outcome when measured with MRI, the study may not be powered to determine the effect of the interventions on all of the secondary outcomes. However, the use of 3 T MRI for all physiological outcomes optimizes accuracy and precision over other methods. Another limitation is the use of clinically administered ultrasound to assess the exploratory outcome of tumor size change in neoadjuvant patients. While ultrasound that is administered as standard of care will be standardized in terms of reporting methods, there may be some variation among different technologists and radiologists. Further, some tumors are ill-defined at baseline or with response to neoadjuvant therapy when assessed by ultrasound and this may reduce accuracy. However this outcome is exploratory and is intended to generate pilot data for further exploration.

## Abbreviations

BNP: b-type natriuretic peptide
CREATE: caloric restriction and exercise protection from anthracycline toxic effects
FACT: functional assessment of cancer therapy
LV: left ventricle
LVEF: left ventricular ejection fraction
MRI: magnetic resonance imaging
REE: resting energy expenditure
RPE: rating of perceived exertion
S_a_O_2_: arterial oxygen saturation
S_v_O_2_: venous oxygen saturation
VO_2_: local volume of oxygen consumption
VO_2_R: whole body volume of oxygen consumption reserve
