# Correction to: Rationale and design of the Caloric Restriction and Exercise protection from Anthracycline Toxic Effects (CREATE) study: a 3-arm parallel group phase II randomized controlled trial in early breast cancer

**DOI:** 10.1186/s12885-019-5298-9

**Published:** 2019-01-15

**Authors:** Amy A. Kirkham, D. Ian Paterson, Carla M. Prado, John R. Mackey, Kerry S. Courneya, Edith Pituskin, Richard B. Thompson

**Affiliations:** 1 Department of Biomedical Engineering, University of Alberta, 1098 ResearchTransition Facility, 8308-114 Street, Edmonton, AB T6G 2V2 Canada; 2 Department of Medicine, Division of Cardiology, University of Alberta, Edmonton, Canada; 3 Department of Agricultural, Food & Nutrition Science, University of Alberta, Edmonton, Canada; 4 Department of Oncology, University of Alberta, Edmonton, Canada; 5 Faculty of Kinesiology, Sport, and Recreation, University of Alberta, Edmonton, Canada; 6 Faculty of Nursing, University of Alberta, Edmonton, Canada

**Correction to: BMC Cancer**

**https://doi.org/10.1186/s12885-018-4778-7**

Following publication of the original article [1], we have been notified that one of the author names was listed incorrectly. Both incorrect and correct author names are presented below.

- Originally published name:
  - John M Mackey
- Correct name:
  - John R Mackey
